# Genetic Research on Neuropsychiatric Disorders and Complex Human Diseases

**DOI:** 10.3390/biomedicines14050950

**Published:** 2026-04-22

**Authors:** Wen Zhang

**Affiliations:** 1 Guangzhou Institute of Science and Technology, Guangzhou 510540, China; zhang.wen81@gmail.com; Tel.: +86-1-713-550-2333; 2 Center for Urban and Environmental Solutions, Charles E. Schmidt College of Science, Florida Atlantic University, Boca Raton, FL 33496, USA

## 1. Defining Disease Interplay and Molecular Signatures

Genetic research on neuropsychiatric disorders and other complex diseases is advancing rapidly. This progress spans diverse conditions, including cancer and autoimmune and aging-related diseases. This Special Issue explores the intersection of genetic factors across these conditions, emphasizing the integration of traditional and modern methodologies. Neuropsychiatric disorders, including schizophrenia, bipolar disorder, and major depression, exemplify the intricate interplay of genetic and environmental influences. Simultaneously, complex diseases like cancer and autoimmune disorders present unique genetic challenges and opportunities for research. By utilizing bioinformatics tools and advanced data analysis methods, researchers can effectively analyze large-scale genomic datasets. A central theme uniting several contributions is the investigation of intricate molecular links governing disease progression and comorbidity. Studies employed integrative multi-omics analysis to elucidate the shared genetic landscape between intestinal system diseases and Colorectal Cancer (CRC) progression [1,2,3]. One comprehensive approach identified 160 shared differentially expressed genes enriched in critical pathways such as cell metabolism, immune homeostasis, inflammation, and gut–brain communication [1]. Furthermore, the analysis uncovered nine key hub proteins [1] including seven upregulated (CD44, MYC, IL17A, CXCL1, FCGR3A, SPP1, IL1A) and two downregulated (CXCL12, CCL5), which serve as potential therapeutic targets and prognostic biomarkers for intestinal system disease-associated CRC.

In the context of complex neurological disorders, another paper utilized whole exome sequencing to explore the genetic heterogeneity underlying neurodevelopmental disorders (NDDs) in four unrelated consanguineous families [4]. This work identified several novel, rare, and ultra-rare variants. These findings were concentrated in key genes, specifically HGSNAT, KDM6B, LMNA, and WFS1. Critically, genetic analysis facilitated the differential diagnosis of one proband, revealing an overlap between a suspected neurodevelopmental disorder and a neurodegenerative condition that caused by a bi-allelic HGSNAT variant [4,5,6].

The meticulous maintenance of neuronal protein homeostasis and its disruption in neurological diseases was explored in relation to Fragile X Syndrome (FXS) [7,8,9]. One study demonstrated an Extracellular Vesicle (EV)-dependent mechanism regulating the intracellular level of the crucial synaptic protein CYFIP2 [7]. Notably, neurons from *Fmr1* KO mice (a model for FXS) exhibited reduced EV secretion and elevated intracellular CYFIP2 levels [7]. Conversely, glutamate treatment effectively reduced CYFIP2 levels by forcing EV release in these FXS neurons. This suggests that impaired EV-mediated export may be a significant factor in FXS-related synaptic deficits.

## 2. Advanced Diagnostics and Prognostic Indicators

Leveraging cutting-edge analytical tools, several studies focused on improving molecular profiling for diagnosis and prognosis. One groundbreaking study utilized Matrix-Assisted Laser Desorption Mass Spectrometry Imaging (MALDI-MSI) to provide a spatial metabolomic map of Hypoxic–Ischemic Encephalopathy (HIE) in neonatal mice [10]. The analysis confirmed severe reprogramming of energy metabolism in the ischemic edema area [11], characterized by a blockade in the tricarboxylic acid cycle (evidenced by the accumulation of metabolites like citrate, succinate, L-glutamate, and glucose). Furthermore, the tissue showed dramatic ATP depletion (reduced by 53% to 73%) and regional specificity in lipid remodeling, including the abnormal accumulation of xanthine in the hippocampus of the affected side. The findings strongly implicate xanthine metabolism, mitochondrial protection, and lipid remodeling as crucial therapeutic targets for HIE.

In cancer prognosis, research on the inflammatory cytokine Resistin (RETN) and lung cancer revealed that high RETN expression in tumor tissues correlated positively with metastasis (particularly distant metastasis) and negatively with overall survival in lung adenocarcinoma [12,13,14,15]. This established RETN expression as an independent predictor of overall survival in lung adenocarcinoma patients. When examining the relationship between RETN polymorphisms (rs1862513 and rs3745367) and the outcomes of platinum-based chemotherapy, one study found no overall association with cancer risk, chemotherapy response, or survival [12]. However, the RETN rs1862513 polymorphism did emerge as a potential biomarker linked specifically to severe gastrointestinal toxicity in treated patients.

## 3. New Avenues for Therapeutic Intervention

Two papers in this issue highlight promising therapeutic compounds capable of modulating fundamental biological processes to combat disease. One study investigated Thymopentin (TP5), a synthetic immunomodulatory pentapeptide, for its potential in cancer immunotherapy [16]. TP5 demonstrated potent, broad-spectrum antitumor efficacy across multiple murine models (melanoma, colorectal carcinoma, hepatocellular carcinoma). Crucially, this effect was strictly T cell-dependent [17], and mechanistically, TP5 promoted thymic rejuvenation, actively increasing T cell populations within the thymus and reversing chemotherapy-induced thymic atrophy. Furthermore, TP5 reprogrammed T cell function, preventing T cell exhaustion (by reducing inhibitory receptor expression like PD-1 and TIM-3) and restoring effector function (by enhancing IFN-γ, TNF-α, and IL-2 secretion). The paper concluded that TP5 acts as a potent adjuvant, synergistically enhancing the efficacy of adoptive T cell therapy.

In neurological health, another study focused on the neuroprotective effects of Edaravone (Eda) in attenuating memory deficits induced by sleep deprivation (SD) in mice [18]. Eda pretreatment successfully improved cognitive impairment in the Lashley III maze paradigm. The mechanism involved multifaceted neuroprotection: Eda exhibited strong antioxidant properties (lowering MDA levels and elevating total antioxidant capacity (TAC), SOD, and GPx activity), countered neuroinflammation (decreasing serum corticosterone and hippocampal IL-1β and TNF-α), and restored synaptic integrity by increasing the expression of synaptic proteins (GAP-43, SYP, SYN-1, and PSD-95) in the hippocampus. These findings promote Eda as a promising candidate for treating neuropsychiatric syndromes associated with SD.

## 4. Outlook

Collectively, the papers in this issue mark significant strides in basic and translational biomedicine. They highlight the necessity of sophisticated, multi-layered analyses to unravel complex pathologies, ranging from the interconnectedness of inflammation and cancer to the fine regulation of synaptic proteins in neurodevelopmental disorders. The insights provided, particularly the identification of novel biomarkers and the mechanistic validation of therapeutic agents like Thymopentin and Eda, offer a robust foundation for improved diagnostic and personalized treatment strategies in the coming years. We congratulate the authors for their impressive contributions to this field and acknowledge the support and dedication of the Academic Editor and the editor(s) in the production of this Special Issue.
